# Pulmonary Lymphomatoid Granulomatosis With Hemophagocytic Lymphohistiocytosis as the Initial Manifestation

**DOI:** 10.3389/fonc.2020.00034

**Published:** 2020-01-29

**Authors:** Li Xu, Xuan Zhang, Ying-Juan Lu, Yan-Hua Zheng, Guang-Xun Gao

**Affiliations:** Department of Hematology, Xijing Hospital, Fourth Military Medical University, Xi'an, China

**Keywords:** lymphomatiod granulomatosis, hemophagocytic lymphohistiocytosis, pathology, rare lymphoma, Epstein-Barr virus

## Abstract

Lymphomatoid granulomatosis (LYG) is an extremely rare angio-centric and angio-destructive B-cell lymphoproliferative disease. Driven by Epstein-Barr virus (EBV), LYG predominantly involves the bilateral lungs. Commonly presenting as multiple nodules in the lung, pulmonary LYG can masquerade as various infectious diseases, vasculitis, lung cancer, or other metastatic neoplasm. It is difficult to be diagnosed and is always neglected by clinicians. No standardized therapeutic regimens for LYG has been established yet now. Hemophagocytic lymphohistiocytosis (HLH), a life-threatening condition caused by abnormal activation of macrophages and T-cells, is characterized by fever, hepatosplenomegaly, pancytopenia, hypercytokinemia, and the presence of hemophagocytosis within the bone marrow, liver, spleen, or other lymphatic tissue. We herein report a 55-year-old man with recurrent fever, severe jaundice, and multiple high-density opacities and nodules in both lungs, who was finally diagnosed with pulmonary LYG (Grade 3) manifested with secondary HLH. Administration of HLH-1994 protocol led to the rapid control of the symptoms caused by HLH. Rituximab-based combination therapy was useful yet LYG (Grade 3) progressed rapidly. This case demonstrates that tissue biopsy is essential for early pathological diagnosis and effective treatment of LYG.

## Introduction

Lymphomatoid granulomatosis (LYG), an extremely rare B-cell lymphoproliferative disease, predominantly involves the bilateral lungs but can also affect skin, central nervous system, kidney, liver, spleen, and other organs. Epstein-Barr virus (EBV) infection plays a pivotal role in the initiation and pathological progression of LYG (1). It is an angio-centric and angio-destructive disorder characterized by abundant reactive T-cells admixed with atypical EBV-infected B cells in a polymorphous inflammatory background (2).

Hemophagocytic lymphohistiocytosis (HLH), a life-threatening condition caused by abnormal activation of macrophages and T-cells, is characterized by fever, hepatosplenomegaly, pancytopenia, hypercytokinemia, and the presence of hemophagocytosis within the bone marrow, liver, spleen, or other lymphatic tissue (3). HLH is divided into two broad categories according to underlying etiologies: primary and secondary. Primary or familial HLH, a rare genetic disease with autosomal recessive inheritance, is caused by pathological mutations in PRF1, UNC13D, STX11, STXBP2, LYST, RAB27A, SH2D1A, BIRC4(XIAP), ITK, AP3β1, MAGT1, and CD27, thus leading to the anergy of nature killer (NK) cells or cytotoxic T-lymphocytes(CTLs) to target cells effectively (4–6). Secondary or acquired HLH may be closely correlated with and triggered by various infection, autoimmune disease and malignancies, among which lymphoma-related HLH and EBV infection-associated HLH (EBV-HLH) are the most common (7–9).

Due to the rarity of LYG, it is often neglected in the differential diagnoses of pulmonary nodules or lesions. Herein, we report a rare case of pulmonary LYG initially manifested with secondary HLH.

## Case Presentation

A 55-year-old man was referred to department of hematology with a 5 month history of recurrent fever, severe jaundice, and fatigue. Thoracic computed tomography (CT) scans in another hospital revealed multiple high-density opacities and nodules in both lungs. Despite the empirical use of cefoperazone and levofloxacin for a suspected pulmonary infection, no significant improvements were achieved both clinically and radiologically. A repeat thoracic CT scan showed exacerbated lung opacities and nodules in both lungs without any cavitations, suggesting the high clinical suspicion for pulmonary tuberculosis. However, antituberculosis medication did not mitigate his symptoms and then discontinued due to the lack of evidence supporting tuberculosis. He denied history of chronic obstructive pulmonary disease, autoimmune disease, hepatitis, and AIDS.

On physical examination, the patient had severe anemic appearance with noticeable jaundice and sporadic petechia and ecchymosis. His temperature was 38.9°C with no enlarged superficial lymph nodes in the cervical, axillary, or inguinal regions. Both the liver and spleen were unpalpable. The rest of the physical examination, including respiratory system, was unremarkable.

The patient's laboratory results indicated bicytopenia (leukocytes 6.93 × 10^9^/L, hemoglobin 53 g/L, and platelets 13 × 10^9^/L) and markedly increased levels of serum ferritin (5,350 μg/L) and β2-microglobulin (13.1 mg/L). Other laboratory tests revealed triglycerides (TG) of 7.95 mmol/L (normal range: 0.56–1.70 mmol/L), fibrinogen 1.13 g/L, lactate dehydrogenase (LDH) 475 U/L, total bilirubin 118 μmol/L (normal range: 3.4–20.5 μmol/L), direct bilirubin 89.4 μmol/L (normal range: 0.0–6.8 μmol/L), albumin 22.8 g/L, alanine aminotransferase (ALT) 120 U/L, aspartate transaminase (AST) 176 U/L, procalcitonin (PCT) 3.48 ng/ml, serum creatinine (Scr) 149 μmol/L (normal range: 57–97 μmol/L). Blood cultures (for aerobic bacteria, anaerobic bacteria and fungi), *T*-spot test (1–3)-Beta-D-Glucan assay (G test), and galactomannan assay (GM test), and cytomegalovirus (CMV) DNA serological tests were all negative. Serological tests for EBV-related antibody showed that EBV-capsid antigen-IgG (EBV-CA-IgG) and EBV nuclear antigen (EBNA)-antibody were positive, while the copy level of EBV-DNA was normal. The absolute count of CD4+T cell was 0.31 × 10^9^/L and the ratio of CD4+T cell to CD8+T cell was 0.76. Autoantibody profile indicated no abnormity. Chest CT scans demonstrated scattered patch-like opacities with a little bilateral pleural effusion and multiple enlarged mediastinal lymph nodes. On transabdominal ultrasound, he had moderate splenomegaly with the spleen 16 cm in length and 5.7 cm in thickness.

**Figure 1:**
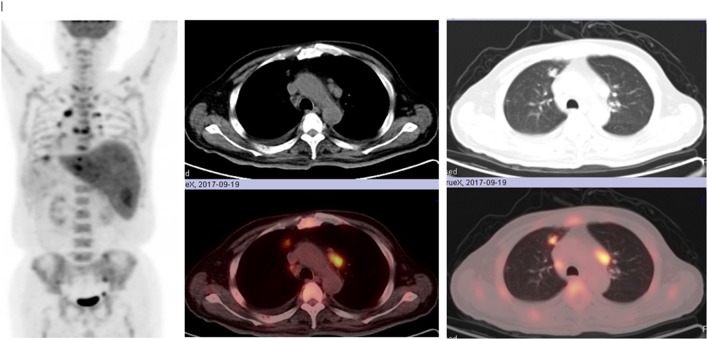
Multiple hypermetabolic nodular lesions in the anterior segment of superior lobe of right lung, middle lobe of right lung, apico-posterior segment of superior lobe of left lung, bilateral pleura, liver, multiple bone lesions in skull, maxillae, bilateral humerus, bilateral clavicle, bilateral scapula, sternum, some vertebrae, some ribs, bilateral femurs, and the pelvis on FDG-PET CT scan.

18F-fluorodeoxyglucose (FDG) positron emission tomography (PET) demonstrated irregularly-shaped hypermetabolic nodular lesions in the anterior segment of superior lobe of right lung with a maximum standard uptake value (SUV) of 5.8 g/ml, suggesting highly clinical suspicions for lung cancer. On FDG-PET CT scan, multiple hypermetabolic nodular lesions with a maximum SUV of 4.7 g/ml were also observed in the anterior segment of superior lobe of right lung, middle lobe of right lung, apico-posterior segment of superior lobe of left lung, bilateral pleura, and the liver (Figure 1). Intense FDG uptake was shown in the lymph nodes of posterior region of left sternocleidomastoid muscle, right internal mammary region, hilums of both lungs and mediastinum. Multiple bone lesions with hypermetabolic FDG uptake (maximum SUV of 5.6 g/ml) were observed in skull, maxillae, bilateral humerus, bilateral clavicle, bilateral scapula, sternum, some vertebrae, some ribs, bilateral femurs, and the pelvis. In a nutshell, the results of PET-CT examination raised highly clinical suspicion for malignant tumor with multiple metastatic sites.

Bone marrow aspirate smears showed that frequently-observed reticulocytes and macrophages were engulfing and phagocytizing nucleated red blood cells and platelets. Bone marrow biopsy revealed an obvious proliferation of lymphoid histiocytes. Only 42.84% TET2 mutations were detected through next generation sequence of bone marrow aspirate, while other gene aberrance associated with hematological malignancies were not detected. Flow cytometry analysis indicated that cells positively expressed CD10, CD20, CD3, CD7, and CD56, dimly expressed CD38 and CD4, and were negative for TCRαβ, TCRγδ, CD33, CD13, lambda, and kappa. Flow cytometry also revealed abnormal lymphocyte subpopulations on which B-lymphocyte antigens were predominantly expressed. The decreased number of CD4+T and the increased number of CD8+T indicated the immunosuppressive state.

Based upon the patient's clinical presentation and laboratory tests, the diagnosis of secondary HLH was made after fulfilling six out of eight diagnostic criteria for HLH-2004 (10), including fever, splenomegaly, bilineage cytopenia, elevated triglyceride (>3 mmol/L), and/or hypofibrinogenemia (<1.5 g/L), elevated serum ferritin (≥500 μg/L), and the presence of phagocytic macrophages and erythrophagocytosis (multiple red blood cells inside the phagocytic macrophage) within the bone marrow. Other supportive evidence for diagnosis of HLH consisted of elevated transaminases, bilirubin, and LDH. But decreased or absent NK cells activity (defective function of NK cells) and soluble interleukin-2 receptor (sCD 25, sIL-2R) levels could not be determined in our hospital. Besides, no HLH-related genetic mutations were detected in this patient.

The patient was started on empirical prescription of meropenem for the treatment of pulmonary infection. After combinative treatment with etoposide and oral dexamethasone (HLH-1994 protocol) (11), the fever soon subsided, jaundice receded, and then the blood cell count, raised triglycerides, liver function, renal function, and coagulation dysfunction gradually recovered to normal levels. However, serum ferritin and β2-microglobulin remained still elevated far above the normal levels. After 1 month of HLH treatment, a repeat PET-CT examination demonstrated that the previous multiple hypermetabolic lesions dramatically shrank with decreased SUV.

**Figure 2:**
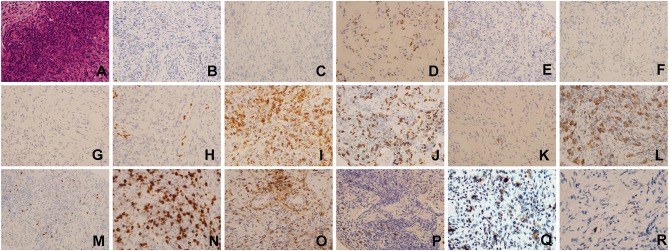
Histopathologic examination and Immunohistochemical staining results of lung biopsy specimen(×400). **(A)** Hematoxylin-eosin (HE) staining, **(B)** AE1/AE3(–), **(C)** ALK1(–), **(D)** CD3(–), **(E)** CK7(–), **(F)** P40(–), **(G)** P63(–), **(H)** TTF(–), **(I)** CD4(–), **(J)** CD8(–), **(K)** CD30(–), **(L)** CD20(+), **(M)** Pax-5(+), **(N)** Ki67(+), **(O)** LCA(+), **(P)** EBER(ISH:EBER-positive cells > 50/HPF), **(Q)** LMP1(+), **(R)** EBNA2(–).

Considering the suspicion for malignant tumor with multiple metastatic sites indicated by PET-CT scan, fiberoptic bronchoscopic lung biopsy was performed to differentiate the diagnosis of tuberculosis, infection, or cancer. Histopathologic examination of lung biopsy specimen demonstrated proliferation of fibrous tissue and infiltration of chronic inflammatory cells interspersed with atypical large B-lymphocytes. Immunohistochemistry results showed that the large atypical lymphocytes stained strongly positive for CD20, LCA, Ki67, Pax-5, latent membrane protein 1 (LMP1), while completely negative for AE1/AE3, ALK1, CD3, CK7, P40, P63, TTF, CD15, CD4, CD8, EBNA2. *In situ* hybridization for EBV encoded small RNA (EBER) was strongly positive in numerous large atypical lymphocytes (>50/high power field) (Figure 2). Ultrasound-guided percutaneous needle aspiration and biopsy of liver showed active liver damage and extramedullary hematopoiesis without evidence of malignancy. Bone marrow biopsy revealed EBER negativity and there was no involvement of cancer cells in the bone marrow. Based on the above histopathologic characteristics, we reached the final diagnosis of pulmonary LYG (Grade 3) with secondary HLH.

The patient was administered with three cycles of R-CDOPE regimen (rituximab in combination with cyclophosphamide, liposomal doxorubicin, vincristine, predisone, and etoposide) and his body temperature eventually decreased to the normal level. After one cycle of R2 regimen (rituximab in combination with lenalidomide) was initiated, clinical symptoms lessened and a follow-up chest CT scans revealed noticeable amelioration of multiple nodular opacities in the lung. And then oral lenalidomide was prescribed as the maintenance therapy. The disease remained stable for 3 months with gradual resolution of lung nodules on chest imaging. However, the patient developed pancytopenia and unfortunately died of disease progression 16 months later after initial presentation.

## Discussion

Primary HLH, secondary HLH induced by infection and autoimmune disease develop predominantly in children and adolescents. While, secondary HLH triggered by malignancy, especially lymphoma, most occurs in adults and the elderly. The prognosis of primary HLH is much worse than secondary HLH (12, 13). Both primary and secondary HLH are characterized by severe cytokine release storm and excessive inflammation (4). The mechanisms of HLH can be summarized as follows. NK cells function by releasing the granules which contain perforin and granzymes. Perforin adheres to the membrane of target cell and drills pores through which granzyme floods into the cell and facilitates the degradation of proteins, thus resulting in the cell apoptosis and suspension of inflammatory cascade (14). Therefore, the granules and perforin released by NK cells play a pivotal role in mediating cytotoxicity and maintaining the homoeostasis of antigen presentation (14, 15). Diminished or absent activity of NK cells and CTLs leads to a decrease in granule release, thus triggering the uncontrolled stimulation of macrophages, sustained inflammatory response and cytokine storm. The hypersecretion of proinflammatory cytokines exacerbate the tissue damage and organ failure (16, 17).

Therapeutic strategies of HLH involve short-term regimen which is oriented to instant control of excessive inflammatory response and long-term regimen which aims at coping with the underlying etiologies while giving the supportive treatment (18, 19). HLH-94 protocol consisted of etoposide and dexamethasone (EP reginmen) for eight cycles of induction therapy followed by allogeneic hematopoietic stem cell transplantation (Allo-HSCT) (11). Etoposide, a topoisomerase II inhibitor, constitutes the mainstay of HLH treatment by inducing apoptosis of activated immune cells, inducing errors in DNA replication and retarding the biosynthesis of EBV nuclear antigen (20, 21). HLH-2004 treatment protocol was a revised version of HLH-94, adding cyclosporine A (CsA) to the EP regimen with the aim of inhibiting hypercytokinemia and undue activation of immune cells (10). However, there exists no evidence to confirm that patients would gain more benefits after initiation of HLH-2004 protocol. Therefore, HLH-2004 protocol is not highly recommended during induction therapy especially when the underlying causes are unknown or the possibility of malignancy-triggered HLH cannot be excluded. That is to say, HLH-94 remains still a reliable and preferred regimen for induction therapy (22).

Despite the advancements with wide application of HLH-94 protocol, ~30% of HLH patients show no response and the disease becomes refractory. Wang et al. initiated a prospective clinical trial which was aimed to explore the efficacy of DEP regimen (liposomal doxorubicin in combination with etoposide and methylprednisolone) as a salvage therapy in adult patients with refractory HLH. The DEP regimen yielded an encouraging overall response rate of 76.2% and was well-tolerated. Their study also indicated that DEP regimen could not only lead to prolongation of patient survival but also bridge the gap from induction therapy to treatment of underlying etiologies (23).

Allogenic hematopoietic stem cell transplantation (allo-HSCT) currently represents the only final solution for both primary HLH and refractory secondary HLH. Despite being recommended in the HLH-94 and HLH-2004 protocol, myeloablative conditioning (MAC) regimens which consisted of etoposide, busulfan, and cyclophosphamide had a close correlation with high transplantation-related mortality and morbidity. Although reduced-intensity conditioning (RIC) regimen was reported of increased survival rate and decreased toxicity compared to MAC, the high incidence of mixed chimerism was inevitable (24). Recently, some studies had suggested that RIC might be more reasonable than MAC for HLH patients (22). Autologous HSCT (auto-HSCT) was regarded more applicable to lymphoma-induced HLH (9). Ironically, transplantation-induced HLH or post-HSCT HLH is a specific entity of secondary HLH and infections, especially EBV or cytomegalovirus infections, are the potential trigger of post-HSCT HLH, the risk of which could be reduced by etoposide-based conditioning regimen (25).

On chest CT, pulmonary LYG generally presents with multiple nodules with irregular, but well-defined margins, which can masquerade as pulmonary infections (especially tuberculosis and pneumonia), interstitial lung disease, vasculitides (Wegener's granulomatosis), sarcoidosis, lung cancer, or metastatic malignancies (26, 27). Nodules or masses with lymphatic distribution represent the lymphoproliferative nature of pulmonary LYG. The nodules distributed along the bronchovascular bundles or interlobular septa are correlated with angioninvasiveness which can be manifested in central low attenuation, ground-glass halo, and peripheral enhancement of the nodules (28).

Owing to the fact that bronchoscopic or percutaneous needle biopsy may sometimes be positive in a small proportion of the patients, tissue specimen obtained by open lung biopsy or video-assisted thoracoscopic surgery (VATS) is required for the definitive diagnosis and differential diagnosis. Polymorphic lymphocytic infiltrate, vasculitis, and granulomatosis with central necrosis are the definitive criteria for pathological diagnosis of LYG, which can be subcategorized into three grades according to the number of EBV-positive large B-cell counted per high-power field (HPF). EBV-positive B cells are infrequently identified in grade 1 LYG, with <5 EBV-positive B cells per HPF while grade 2 reveals 5–20 cells per HPF. Grade 3 shows abundant EBV-positive B cells by *in situ* hybridization of EBER, numbering more than 50 cells per HPF (26). Therefore, the final diagnosis of this patient corresponded to pulmonary LYG, grade 3.

Both primary and secondary Immunocompromised individuals with chronic or latent EBV infection are predisposed to develop LYG or other lymphoproliferative diseases probably because defects in cellular immunity can severely retard EBV eradication and cannot inhibit the proliferation of EBV-infected B-cells (29, 30). It has been reported that patients with EBV infection are likely to have abnormal T lymphocyte subpopulation, indicating the state of immune-disregulation. LYG may occur accompanied by various kinds of autoimmune diseases, such as Sjogren syndrome and rheumatoid arthritis (31, 32).

Neither standard regimen nor consensus for LYG treatment has been established up till now. Based upon the number of EBV-infected B cells by ISH, therapeutic strategies have varied from “watch and wait” or steroids alone to intense immunochemotherapy. Grade 1 and 2 LYG may be treated with steroids alone, interferon-α2b and rituximab (33, 34). Grade 3 LYG, characterized by rapid progression, bears a striking resemblance to diffuse large B cell lymphoma (DLBCL) both clinically and pathologically. To some extent, grade 3 LYG is considered a rare subtype of DLBCL and should be treated in the same manner with aggressive chemotherapy (26). Considering the histological diagnosis of this patient, treatment was initiated with rituximab-based combination therapy. It was reported that a patient with refractory pulmonary LYG was successfully treated by auto-HSCT (35). Despite various therapeutic approaches, LYG has a bleak prognosis with a median survival of <2 years and 5-year mortality of 60–90% (1).

## Conclusion

This case demonstrates that LYG in the lung can mimic many common pulmonary lesions including tuberculosis both clinically and radiographically, which poses a great challenge for clinicians. When a patient does not respond well to empirical therapy, we should come up with this rare lymphoma and high priority should be given to pathological diagnosis through tissue biopsy. The patients with LYG can present HLH as an initial manifestation. Especially, LYG patients who have fever, hepatosplenomegaly, pancytopenia, abnormal liver function should be evaluated for HLH. Early diagnosis and effective treatment according to precise stratification may extend the patient's survival.

## Data Availability Statement

The raw data supporting the conclusions of this article will be made available by the authors, without undue reservation, to any qualified researcher.

## Ethics Statement

The studies involving human participants were reviewed and approved by the Ethics Committee of Xijing Hospital affiliated to Fourth Military Medical University. The patients/participants provided their written informed consent to participate in this study. Written informed consent was obtained from the individual(s) for the publication of any potentially identifiable images or data included in this article.

## Author Contributions

Y-HZ and LX gathered the clinical information and drafted the manuscript. G-XG and Y-HZ approved the final diagnosis and formulated the therapeutic strategies. G-XG and XZ acquired and interpreted the radiographic results. Y-JL and XZ performed the tissue biopsy and described the pathological findings. All authors critically revised and gave the final approval of the manuscript.

### Conflict of Interest

The authors declare that the research was conducted in the absence of any commercial or financial relationships that could be construed as a potential conflict of interest.
